# How Memory Counts in Mathematical Development

**DOI:** 10.5334/joc.248

**Published:** 2023-01-06

**Authors:** Ilse E. J. I. Coolen, Julie Castronovo

**Affiliations:** 1 Department of Psychology, University of Hull, Cottingham road, Hull, HU67RX, UK; 2 Université de Paris, LaPsyDÉ, CNRS, F-75005 Paris, FR

**Keywords:** Numerical Cognition, Memory, Development

## Abstract

Memory has been well-established as a predictor of mathematics achievement in child development. Nevertheless, empirical evidence remains elusive on the unique role of the different forms of memory and their specific mechanisms as predictors of mathematics development. Therefore, in this study, the role of visuospatial short-term memory, visuospatial working memory, verbal short-term memory, and verbal long-term memory was investigated at three key stages of the development of mathematics (5–6 years, 6–7 years, 7–8 years), as well as their interactions across development. The relation between the different memory types and informal and formal mathematics was also studied. The findings of this study provide empirical support for a shift in the relation between different memory types and mathematics achievement over development with: 1) visuospatial short-term memory predicting informal mathematics achievement at the age of 5–6 years; 2) visuospatial working memory predicting informal and formal mathematics achievement at the age of 6–7 years; and 3) verbal short-term memory predicting formal mathematics achievement at the age of 7–8 years. These shifts clearly appear consistent with children’s mathematics curriculum content over time and the requirements of mathematics acquisition at specific stages in development. With these findings, the unique role of various forms of memory in the development of mathematics and the timeframe in which they play a crucial part is highlighted, which should be taken into consideration for future research and possible intervention studies in children’s mathematics achievement.

## Introduction

Children’s mathematics achievement has a complex development with a large number of identified predictors which are thought to form some foundational basis for mathematics understanding (e.g., visuospatial short-term memory, verbal intelligence, phonological abilities and executive functions such as working memory, inhibition and shifting, Bull et al., 2008; Passolunghi et al., 2014, 2015; Purpura et al., 2017). These predictors are often pitted against each other to identify the most important early skills in mathematics development, aiming to find early skills that could be used as part of interventions to improve mathematics. This renders it more difficult to interpret contradictory findings in the literature and to generate theories on how predictors and their particular mechanisms may contribute to mathematics achievement development in children. Nevertheless, some studies have focused on interactions between specific predictors at various stages in a child’s development in order to establish a more in depth understanding of the role of these predictors (Allen et al., 2020; De Smedt et al., 2009; van der Ven et al., 2013). One line of literature has specifically looked at the role of memory in mathematics achievement and found interaction between different memory types such as verbal compared to visual memory (Allen et al., 2020; De Smedt et al., 2009). Therefore, this study is on memory and explored the different memory types which have been previously identified as predictors of mathematics achievement. It aimed at replicating and expanding on prior theories of their role in mathematics development. The different memory types examined were: short-term memory, long-term memory, and working memory. All three types have previously been related to mathematics achievement in early primary school development (Allen et al., 2020; Bull et al., 2008; De Smedt et al., 2009) and the proposed mechanisms with regards to their respective contribution to the development of mathematics will be described.

### Short-term memory

When learning the number sequence, children learn to compare one quantity or number to a just previously presented quantity or number, requiring short-term memory. The relation between short-term memory and mathematics achievement has been repeatedly examined in the literature with an emphasis on visuospatial skills (Fanari et al., 2019; Holmes & Adams, 2006; Sella et al., 2016). Visuospatial skills (i.e. maintenance and manipulation of visuospatial information such as those measured through block span tasks where participants have to reproduce a given spatial sequence of block taps) have been suggested to play a significant role in early mathematical development in children, when children rapidly acquire new number skills, such as counting and associating small quantities to abstract numerical concepts (i.e., Arabic digits, number words; Holmes et al., 2008a). Indeed, when the child is learning to count, a placeholder structure for numbers is created along a mental number line in a fixed order (small numbers on the left and larger numbers on the right; Gevers et al., 2010; Holmes et al., 2008a). Even prior to formal schooling, pre-schoolers use visuospatial strategies to learn the counting sequence and place small numbers on a spatial mental number line, illustrating how visuospatial skills can be important to early number skills (Bull & Lee, 2014). Even after formal schooling takes place, simple calculations can require visuospatial skills (Holmes et al., 2008b), as it has been suggested that children use their visual mental number line in order to add numbers together. For instance, young children prefer to count on their fingers during additions, increasingly adding steps, which could be to visualise their mental number line (Fabbri & Guarini, 2016). More support for the link between spatial skills and mathematics through using the mental number line, was found by Gunderson et al. (2012), demonstrating that spatial skills help children to acquire a linear spatial representation of numbers, which in turn predicts symbolic calculations. However, this association between visuospatial skills and mathematics achievement has been found to decrease with age, coupled with an increase in the association between verbal memory and mathematics (De Smedt et al., 2009; Holmes et al., 2008b, but see Atit et al., 2022). This suggests that gradually children step back from using visuospatial strategies and begin using the verbal abstract representations more during calculations (De Smedt et al., 2009; Holmes et al., 2008b; Holmes & Adams, 2006). Indeed, De Smedt et al. (2009) found specific support for a switch around the ages of 6 and 7 years old, where in the first year of formal schooling in Belgium, visuospatial memory (not verbal memory) was predictive of mathematics achievement, while verbal memory (not visuospatial memory) was predictive of mathematics achievement in the second year of formal schooling (age 7 years). It is suggested that children start using verbal strategies and retrieval around this age.

### Long-term memory

If indeed verbal memory is increasingly important in mathematics development in early primary school because children progressively adopt more verbal strategies, it appears that these acquired verbal strategies do not only rely on short-term memory, but also on long-term memory (i.e., storage and retrieval of arithmetical facts). Although less explored as a predictor of overall mathematics achievement, long-term verbal memory can easily be related to arithmetic development. Indeed, the responses to simple calculations gradually get stored in memory so that they require less time to be solved (Jensen & Whang, 1994). It could therefore be assumed that long-term verbal memory rather than short-term verbal memory would become a better predictor of mathematics achievement once verbal responses have been stored in long-term memory, such as around the age of 7–8 years when arithmetic (e.g., calculation tables) is important in the educational curriculum.

### Working memory

Finally, solving complex mathematical calculations requires active maintenance and simultaneous manipulation of numerical information demonstrating the link between working memory and mathematics achievement. In the development of mathematics, manipulation of symbolic numerical representations is required when performing more complex arithmetic problems that cannot simply be resolved by reproducing stored memories (e.g., subtractions, divisions, and fractions). In line with the increasing complexity of mathematics with development, working memory might become progressively more important as a predictor of mathematics achievement than short-term memory (Best & Miller, 2010). Indeed, prior research found that children require more working memory as the demands of academic achievement enhance (Allen et al., 2020; Geary et al., 2012). While short-term memory tasks rely on maintaining and reproducing information (such as measured in a forward span task), working memory tasks also require the manipulation of said information (such as in backward span tasks) (Aben et al., 2012). Bull et al. (2008) previously found that visuospatial short-term memory, measured with a forwards span task, was a high predictor of mathematics achievement at the first year of primary school in the UK (5–6 years), while visuospatial working memory, measured through a backwards span task, predicted mathematics achievement only at the end of the third year of primary school (7–8 years). Taken together, with these findings support was found that working memory would become progressively more important with age, reflecting the increasing complexity of mathematical calculations.

In light of these prior results, the aim of this study is first to try to replicate earlier findings on the various memory types and their respective role in early primary school mathematics development (i.e., ages 5 to 8 years), and second to expand on these findings to further understand the unique roles of various memory types in mathematics development for example by investigating their roles in formal and informal mathematics achievement. Findings to be replicated include: a) the assumption that various memory types are not equally predictive of mathematics achievement at different ages throughout early development (exploring predictors per age group), especially since all of them do not have the same development rate; b) whether shifts in memory predictors of mathematics achievement can be found across different ages (exploring interactions between memory types and age groups (Year 1, Year 2 or Year 3)).

Findings to be expanded include investigating the mechanisms of how memory is related to mathematics achievement, by a) concurrently studying visuospatial and verbal memory types and b) studying their roles in formal and informal mathematic achievement. Moreover, to further expand the existing findings in the literature, this study includes a verbal short-term memory task that is not a digit span task (i.e., repeating a sequence of spoken numbers) as previously used in the literature (Bull et al., 2008; De Smedt et al., 2009). This was done since it can be assumed that a digit span is closely related to formal mathematics, due to the use of number digits rather than words. By not using a digit span, the current study can investigate whether the link between verbal memory and mathematics still holds when not using numerical information such as digits. Furthermore, in this study a long-term verbal memory task was added to the testing batterie as long-term verbal memory skills might be more important in the oldest age group in this study (7–8 years, Year 3). Indeed, the storage and retrieval of arithmetic facts becomes increasingly more important in the development of mathematics. Finally, the current study also investigated the unique roles of different memory types in both formal and informal mathematics, rather than mathematics achievement as a whole. The distinction between informal (i.e., numerical skills acquired by children outside of the context of formal schooling) and formal mathematics (i.e., acquired through formal schooling) achievement can be controversial since it is difficult to define. Nevertheless, Ginsburg and Baroody (2003) designed the Test of Early Mathematics Ability (TEMA-3), which includes informal (e.g., counting and understanding that adding items results in more items) and formal items (e.g., writing number symbols and arithmetic), allowing us to measure both formal and informal mathematics achievement in children. It is also important to note that this distinction between formal and informal mathematics achievement has previously been successfully used in the literature (Libertus et al., 2013, 2016).

The hypotheses for this study are that a shift should be found in terms of importance of memory predictors of mathematics achievement, with visuospatial short-term memory being a good predictor of mathematics achievement until the age of 6 (Bull et al., 2008; De Smedt et al., 2009), verbal short-term memory should then increase in importance as predictor of mathematics achievement (De Smedt et al., 2009), closely followed by long-term verbal memory and visuospatial working memory (reflecting the increased complexity of mathematical operations and problem solving) from the ages of 7–8 years old (Bull et al., 2008). In addition, expected findings also include that while visuospatial short-term memory should be related to informal mathematics achievement more than formal mathematics achievement (as suggested by Holmes et al., 2008a), immediate and long-term verbal memory and visuospatial working memory should be related to formal mathematics achievement more than informal mathematics achievement (as suggested by De Smedt et al., 2009).

## Method

### Participants

133 children aged 5 to 8 years (the first 3 years of formal primary school education) took part in this study (65 females, 68 males, M_age_ = 83.29 months, SD_age_ = 8.16 months). Descriptive statistics of the children per age group (Year 1: 5–6 years; Year 2: 6–7 years; Year 3: 7–8 years) are presented in Table 1. The children had been recruited from 3 schools in East Yorkshire as part of a longitudinal study on domain-general and domain-specific predictors of mathematics achievement. In the second wave of testing the children (corresponding to the data used in this study), some tasks assessing different memory types were added to the testing batterie to explore the current research questions. The sample size was dependent on the number of participants previously tested in the first wave as part of the longitudinal study not having dropped-out or changed school. Nevertheless, power analysis with alpha = 0.05, power = 0.8, showed that 34 participants were needed for a moderate to large effect size with 6 predictors in a regression analysis, which was obtained in this study. Of the 133 children, 6 did not complete any of the memory measures required for this study. Their data was dismissed. An information sheet was handed to the parents with an option for their child(ren) to opt out from the study at any time. The study received ethical approval from the Ethics Committee of the School of Life Sciences, University of Hull. The anonymised data that support the findings of this study and the analysis script including power analysis have been made available on OSF and can be accessed at https://osf.io/jked2.

**Table 1:** Descriptive statistics for all tasks per age group.


	YEAR 1 (N = 36; 16 MALES)			YEAR 2 (N = 49; 25 MALES)			YEAR 3 (N = 42; 24 MALES)		
		
	M	SD	RANGE	M	SD	RANGE	M	SD	RANGE

Age (months)	74.03	3.09	68–80	82.33	3.83	75–88	92.24	4.30	86–105

TEMA (raw)	35.86	7.85	17–52	44.51	9.66	29–70	56.02	11.41	32–43

Informal TEMA	27.06	4.93	15–34	31.98	3.25	23–38	35.45	3.52	25–43

Formal TEMA	6.97	2.84	2–14	11.24	6.04	4–24	18.12	6.97	6–29

Pathspan forwards	4.35	1.17	2–7	4.72	1.15	2–7	5.45	0.99	4–8

Pathspan backwards	3.69	1.4	2–6	4.3	1.04	2–7	4.43	0.99	2–6

Recall	17.28	5.46	7–29	19.12	5.74	11–30	22.29	7.17	6–39

Delayed Recall	7	2.61	1–13	8.34	2.50	3–15	9.62	2.39	5–15

Pattern Construction	24.86	3.66	18–32	25.61	2.97	20–32	28.21	2.79	22–35

Word Definition	10	4.64	3–18	12.82	3.97	4–19	15.60	4.70	5–23

BAS mean %	48.67	10.03	29–65	53.45	7.44	39–69	60.83	7.31	45–73


#### Procedure

Children were tested individually in a quiet area in the school. The complete assessment time at wave 2 (including tasks not analysed in this study) was approximately 1 hour and 10 minutes, split into 3 sessions. After every session, children received a reward in the form of a sticker. The order of the tasks was the same for all participants to ensure the same testing experience for all children. Instructions were presented orally by the examiner.

### Material

#### Mathematics achievement

Children’s mathematics achievement was measured using form B (form A was used in the first wave of testing) from the Test of Early Mathematics Ability-third edition (TEMA-3; Ginsburg & Baroody, 2003). TEMA-3 allows for overall raw and standardised scores of mathematics achievement, as well as specific scores of informal and formal mathematics achievement, to be measured in children aged 3 to 8 years. This mathematics test, commonly used in the literature (Chu et al., 2016; Devlin et al., 2022; Libertus et al., 2013, 2016), covers a variety of numerical and mathematical skills, such as numbering skills (i.e., counting), number comparison, number literacy (i.e., reading and writing numbers), arithmetical facts, calculation skills and understanding of numerical/arithmetical concepts. The test consists of 72 questions in total with the test ending after 5 consecutive incorrect responses. 40 questions are informal mathematics questions and 32 are formal mathematics questions.

#### Visuospatial short-term memory

To assess visuospatial short-term working memory, the Pathspan, an adaptation of the Corsi block tapping task on Ipad, was used (developed by Hume and Hume, 2012). Nine buttons appear on screen in a pseudorandom pattern. The buttons flash for 1,000 ms per button in a certain pattern until a tone is heard. The child’s task is to remember and reproduce the flashed sequence by touching the buttons on the screen in the same order. The length of the flashed sequences increases in difficulty, starting with two flashed buttons up to nine flashed buttons. Every length is presented three times with a different order to the sequence and the experiment ends with three incorrect responses on the same length. The score for this task corresponds to the highest sequence length achieved with at least one correct response.

#### Visuospatial working memory

The backwards version of the Pathspan task was used to measure visuospatial working memory. The same procedure as in the visuospatial short-term memory Pathspan task was used, except that children were asked to reproduce the sequence of flashed dots backwards. The experiment terminated after children failed the recreate the backwards sequence of the same length three consecutive times. The score obtained in the backwards Pathspan was the highest sequence that the child successfully recreated at least once.

#### Verbal short-term memory and verbal long-term memory

Verbal short-term memory and long-term memory were assessed with the verbal version of the Recall of Objects task from the British Ability Scales – third edition (BAS-3). The Recall of Object task consisted of a page size A4 with 20 pictures of objects. The card was presented face down to the child and the child was instructed to look at all the pictures carefully when the card was turned around and to try and remember as many of the pictures as possible. When the card was then turned over again, the child was asked to name all the objects remembered. The task consisted of 3 consecutive trials with immediate recall and one trial with delayed recall. Trial 1 of the immediate recall was comprised of 40 second exposure time followed by 60 seconds recall time, while trial 2 and 3 consisted of 20 seconds exposure time and 40 second recall time each. The delayed recall trial was administered approximately 20 minutes after the immediate recall trials (as defined in the BAS-3), depending on the administration time of the TEMA-3, which was administered in between the immediate and the delayed Recall of Objects. The delayed recall of objects trial consisted of 60 seconds of recall time of the same pictures of the immediate recall for the child, with no exposure time. The verbal short-term memory score was the total number of objects recalled over the 3 trials. The long-term memory score was the total number of objects recalled after approximately 20 minutes of the last exposure time.

#### Intelligence subscales

Two intelligence subscales of the BAS-3 were used in order to control for general intelligence: 1) Pattern Construction; and 2) Word Definition. The aim of the Pattern Construction task was for the child to reproduce patterns presented on pictures, using two-coloured foam squares or two-coloured blocks within a time frame ranging from 10 to 120 seconds. The test ended after 4 incorrect responses in a set of 5 consecutive items. The subtest Word Definition of the BAS-3 was used to assess verbal intelligence. Children were orally presented with a series of words ordered from easy (e.g., bed) to more difficult (e.g., counterfeit), and were asked to explain the meaning of these words. The task was ended after 5 consecutive incorrect responses.

## Results

### Preliminary analyses

A limited amount of data was missing, with 2 participants missing data on the forwards Pathspan, 4 participants (including the 2 participants with missing data on the forwards Pathspan) had missing data on the backwards Pathspan and 4 different participants had missing data on the delayed Recall task. Little’s test for data missing completely at random was not significant *X*^2^(23, *N* = 127) = 26,97, *p* = .257, indicating that missing data were missing at random and were hence estimated using linear interpolation rather than deleted, using the package ‘imputeTS’ (Moritz, 2018). Table 1 displays all tasks’ mean, standard deviation and range per age group (Year 1: 5–6 years; Year 2: 6–7 years; Year 3: 7–8 years). Table 2 shows a Spearman (due to the use of ordinal Pathspan scores) correlation matrix for all tasks used in the study. As expected, all measures were positively correlated. All predictors were highly correlated to all forms of mathematics achievement (overall, informal and formal TEMA scores).

**Table 2:** Spearman correlations between all tasks measured (N = 127).


		1.	2.	3.	4.	5.	6.	7.

1. TEMA		___						

2. Informal	r	.92	___					

	*p*	<.001						

3. Formal	r	.94	.87	___				

	*p*	<.001	<.001					

4. Pathspan forw.	r	.47	.47	.46	___			

	*p*	<.001	<.001	<.001				

5. Pathspan back.	r	.44	.48	.41	.35	___		

	*p*	<.001	<.001	<.001	<.001			

6. Recall	r	.38	.36	.41	.24	.19	___	

	*p*	<.001	<.001	<.001	.008	.036		

7. Delayed Recall	r	.30	.30	.32	.29	.20	.48	___

	*p*	<.001	<.001	<.001	=.001	=.025	<.001	

8. BAS	r	.60	.61	.59	.37	.43	.50	.29

	*p*	<.001	<.001	<.001	<.001	<.001	<.001	<.001


### Regression analyses

In order to replicate previous findings concerning a shift between visuospatial and verbal memory (De Smedt et al., 2009), mixed effect regression models were carried out, using the ‘nlme’ package in R to explore the variance in overall mathematics scores accounted for by each memory type at different education levels (i.e., per year group). Per age group, in the first step age, Pathspan forward, Pathspan backward, Recall and Delayed Recall were entered as fixed predictors of overall TEMA-3 scores and school was entered as random factor. In a second step, mean BAS scores were entered to control for general intelligence. In all year groups, the regression model controlled for mean BAS scores fitted the data better than the non-controlled model, although not significantly in Year 2, based on the AIC model selection (Year 1: AIC = 249.68, AIC_BAS_ = 247.71, *p =* .046; Year 2: AIC = 358.51, AIC_BAS_ = 356.97, *p =* .060; Year 3: AIC = 312.99.68, AIC_BAS_ = 310.77, *p =* .040.

Standardised regression coefficients for the controlled (controlled for mean BAS) and non-controlled model for Year 1 (5–6 years) are presented in Table 3. The only significant predictor of overall TEMA-3 scores in Year 1 is Pathspan forward. Table 4 shows the BAS controlled and non-controlled mixed regressions predicting overall TEMA-3 for Year 2 (6–7 years). Although Pathspan backward is the only predictor in the non-controlled model, this is not significant in the BAS controlled model. Finally, as can be seen in Table 5, in the model predicting TEMA-3 in Year 3, Recall is the only significant predictor, but only in the model not controlled for BAS. Thus, the unique predictors per age group are: visuospatial short-term memory in Year 1, visuospatial working memory in Year 2 (only without controlling for intelligence) and verbal short-term memory in Year 3 (only without controlling for intelligence).

**Table 3:** Summary of regression analysis for variables predicting overall TEMA-3 in Year 1 (N = 36).


PREDICTORS	MODEL 1			MODEL 2 (BAS CONTROLLED)		
	
	BETA	T	*P*	BETA	T	*P*

Age (months)	–0.81	–0.21	.832	–0.23	–0.61	.548

Pathspan forwards	3.24	2.94	**.007**	2.74	2.50	**.019**

Pathspan backwards	1.57	1.48	.151	0.15	0.12	.906

Recall	0.26	1.08	.288	–0.04	–0.15	.884

Delayed Recall	–0.09	–0.16	.872	–0.08	–0.15	.881

BAS	/	/		0.31	1.84	.077


**Table 4:** Summary of regression analysis for variables predicting overall TEMA-3 in Year 2 (N = 49).


PREDICTORS	MODEL 1			MODEL 2 (BAS CONTROLLED)		
	
	BETA	T	*P*	BETA	T	*P*

Age (months)	0.78	2.34	**.024**	0.66	2.02	.050

Pathspan forwards	–0.38	-0.34	.739	-0.40	-0.36	.718

Pathspan backwards	2.84	2.17	**.039**	2.46	1.89	.066

Recall	0.38	1.57	.124	0.20	0.80	.431

Delayed Recall	–0.16	-0.30	.763	-0.16	-0.30	.768

BAS	/	/		0.33	1.79	.081


**Table 5:** Summary of regression analysis for variables predicting overall TEMA-3 in Year 3 (N = 42).


PREDICTORS	MODEL 1			MODEL 2 (BAS CONTROLLED)		
	
	BETA	T	*P*	BETA	T	*P*

Age (months)	0.62	1.60	.118	0.59	1.58	.123

Pathspan forwards	2.67	1.62	.115	2.52	1.57	.125

Pathspan backwards	2.70	1.77	.085	2.03	1.34	.190

Recall	0.53	2.20	**.035**	0.42	1.73	.093

Delayed Recall	–0.57	–0.82	.419	–0.54	–0.79	.432

BAS	/	/		0.40	1.93	.063


Note: * *p* < .05, **p < .01, ***p < .001, ^1^ *p* = .085, ^2^ *p* = .093, ^3^ *p* = .062.

The second part of the analyses were mixed model regressions of all predictors with the complete data set (3 age groups together) to explore whether the shifts of predictive memory types between different years were also presented in the form of significant interactions. Furthermore, two regressions were also conducted to investigate whether the pattern of predicting memory types differ when predicting formal and informal TEMA-3 scores separately.

Since the main predictor of interest in the following regressions was year group, age was not included in these regressions. Therefore, the age of the children was controlled for in the form of year group rather than age in months. In a first step, the basic three models (1. predicting overall TEMA-3; 2. predicting informal TEMA-3; 3. predicting formal TEMA-3) without interaction terms had the following predictors: BAS, Pathspan forward, Pathspan backward, Recall, Delayed Recall and Year as fixed factors and school as random factor. Table 6 presents the standardised regressions coefficients for the basic three models predicting overall TEMA-3, informal TEMA-3 scores and formal TEMA-3 scores without interactions.

**Table 6:** Summary of regression analyses for variables predicting overall, informal and formal TEMA-3 scores without interactions (N = 127).


PREDICTORS	MODEL 1 OVERALL			MODEL 2 INFORMAL			MODEL 3 FORMAL		
		
	BETA	T	*P*	BETA	T	*P*	BETA	T	*P*

Year 2	5.13	2.69	**.008**	3.43	4.60	**<.001**	2.43	2.12	**.036**

Year 3	12.19	5.35	**<.001**	4.96	5.57	**<.001**	6.88	5.04	**<.001**

Pathspan forwards	1.72	2.41	**.018**	0.74	2.64	**.009**	0.76	1.77	.079

Pathspan backwards	1.97	2.52	**.013**	0.68	2.24	**.027**	0.96	2.05	**.043**

Recall	0.33	2.29	**.024**	0.04	0.73	.465	0.23	2.66	**.009**

Delayed Recall	–0.29	–0.86	.392	–0.06	–0.42	.674	–0.10	–0.47	.636

BAS	0.31	2.90	**.004**	0.17	4.10	**<.001**	0.15	2.34	**.021**


Interactions that were of interest were then added to the three basic regression models. In the first instance, 2-way interactions between the memory predictors and year were added (Pathspan forward*year; Pathspan backward*year; Recall*year; Delayed Recall*year). Subsequently 3-way interactions of interest were added to replicate previous findings and to test the hypotheses of this study of shifts between different memory types (Pathspan forward*Pathspan backward*year: Pathspan forward*Recall*year; Recall*Delayed Recall*year).

For the model predicting overall TEMA-3 scores, only the interaction between Pathspan backward and Year 2 was significant, *t*(111) = 2.06, *p* = .04, indicating that children’s skills in visuospatial working memory, assessed with the Pathspan backward, became significantly more predictive of overall mathematics achievement from Year 1 to Year 2. All other 2-way interactions did not predict overall TEMA-3, *p_s_* > .065. The only significant 3-way interaction predicting overall TEMA-3 was Pathspan forward*Recall*Year 2, *t*(100) = 2.09, *p* = .039. This provides further support that there appears to be a switch in Year 2 between visuo-spatial short-term memory and verbal short-term memory in their role in predicting mathematics achievement in children.

For the model predicting informal TEMA-3 scores, no 2-way interactions were significant, *p_s_* > .073 and no 3-way interactions were significant, *p_s_* > .065.

Finally, for the model predicting formal TEMA-3 scores, there were two significant 2-way interactions: 1) Pathspan forward*Year 2, *t*(111) = –2.27, *p* = .025, showing that visuospatial short-term memory is a worse predictor of formal TEMA-3 in Year 2 compared to Year 1; 2) Pathspan backward*Year 2, *t*(111) = 2.15, *p* = .033, demonstrating that visuospatial working memory is a better predictor of formal TEMA-3 in Year 2 compared to Year 1. No 3-way interactions significantly predicted formal TEMA-3, *p_s_* > .109.

## Discussion

The aim of the current study was to understand the specific role of different memory types in the development of mathematics achievement in primary school children. Although several types of memory have been found to be predictive of mathematics achievement (e.g., Allen et al., 2020; Bull et al., 2008, De Smedt et al., 2009), clear empirical knowledge of their particular mechanisms and involvement in mathematics development has been missing in the current literature. To this end, the current study aimed at exploring the expected shifts between known memory predictors of mathematics achievement based on suggestions made in the literature. Therefore, visuospatial short-term memory, visuospatial working memory, verbal short-term memory and long-term verbal memory were tested in UK-children at the ages of 5–6 years, 6–7 years and 7–8 years. To further explore the specific role of each memory type in children’s development of mathematics achievement, their respective predictive roles to informal and formal mathematics achievement were also explored. This study provided clear empirical support that different forms of memory play unique roles in the development of mathematics achievement. Replicating previous findings by De Smedt et al. (2009), visuospatial short-term memory was found to predict mathematics achievement at the age of 6–7 years, while verbal short-term memory does not. However, after the age of 6–7 years a shift takes place where verbal short-term memory appears to be a predictor of mathematics achievement, while visuospatial short-term memory no longer is. Note that this shift was replicated using both a visuospatial short-term and a visuospatial working memory task and that the verbal short-term memory task used in the current study did not involve numerical information such as digits as used in previous research, hence expanding the current literature by showing that the link between verbal short-term memory and mathematics does not only hold for verbal memory including numerical information (Bull et al., 2008; De Smedt et al., 2009).

Furthermore, the present findings contribute to the literature by extending the current knowledge even further by demonstrating that, although all memory types are closely related, they also have unique roles in mathematics development in children, reflecting the different stage in mathematical development (i.e., year group) and in line with how mathematics are taught at different ages (i.e., informal vs. formal mathematics). Visuospatial short-term memory was hypothesised to be a predictor of mathematics achievement before the age of 6 years (i.e., at the start of formal schooling; Holmes et al., 2008a), which runs parallel to a curriculum that still partly consists of informal mathematics as defined by the TEMA-3 (Ginsburg & Baroody, 2003). Indeed, the current findings demonstrate the importance of visuospatial short-term memory in informal mathematics achievement, but not or to a lesser extent in formal mathematics achievement. With these results it is indeed suggested that visuospatial short-term memory is involved at the early stages of a child’s mathematical development, for example, when skills such as counting and mapping magnitudes unto symbolic abstract number concepts are acquired (Holmes et al., 2008a). This supports the assumption that children use their visuospatial short-term memory to represent numbers on a mental number line when acquiring and developing their counting skills at the start of their education (Bull & Lee, 2014).

On the other hand, visuospatial working memory seemed to be an important predictor of both informal and formal mathematics achievement, mainly in children aged 6–7 years. At this age, children in the UK are required to get more proficient with calculations and their skills in mathematics have to progressively rely more on complex cognitive resources, such as manipulating numerical information. The findings of this study suggest that strategies to solve numerical problems rely more on visuospatial working memory at this stage in the development until some numerical facts become more abstract and are stored in the verbal memory. Retrieving these facts from verbal memory gradually gets easier and faster than having to use complex and slow visuospatial working memory strategies (De Smedt et al., 2009). This is demonstrated by the findings in this study where verbal short-term memory only appears to be a predictor of mathematics achievement from the age of 7–8 years. Furthermore, as expected verbal short-term memory is only predictive of formal mathematics achievement, but not informal mathematics achievement. Indeed, to store numerical and arithmetic facts in long-term memory requires frequent exposure to these facts. This frequent exposure to numerical and arithmetic facts is usually obtained in school and thus through formal mathematics education and not informally learned.

Finally, long-term verbal memory was not predictive of mathematics achievement at any age. Such results are likely to be due to the underlying complexity of long-term memory itself and its different types (i.e., semantic vs. episodic explicit long-term memory). Indeed, the standardised long-term memory task used in the current study measured children’s episodic long-term memory skills, with the need for them to retrieve stored information about a list of objects presented to them 20 minutes earlier (involving components of context in which the memory was formed; Bouyeure & Noulhiane, 2020; Tulving, 1985). On the other hand, arithmetical facts have been repeatedly found to be stored in semantic memory (De Smedt, 2016; Whetstone, 1998), which is automatic and does not require conscious recollection (Bouyeure & Noulhiane, 2020; Tulving, 1985). Therefore, the use of a semantic memory task (e.g., word fluency test; Kormi-Nouri et al., 2003) would be better suited in future studies in assessing whether verbal long-term memory is a significant predictor of mathematics achievement in children.

To sum up, the findings of this study provide further empirical support for several prior suggestions made in the literature. First, a child uses visuospatial short-term memory early in the development of mathematics (5–6 years old; at the start of formal education) when learning to count through using spatial strategies and map magnitudes unto a mental number line. Second, whilst the child is progressively introduced to more formal mathematics in their education, including simple calculations including the manipulation of learned numerical information (e.g., the counting sequence), visuospatial working memory then plays a bigger role in the development of mathematics achievement around the age of 6–7 years. Verbal strategies and arithmetical retrieval then progressively become more adaptive and faster strategies when learning more advanced arithmetic and formal mathematics. This is visible at the age of 7–8 years when verbal short-term memory becomes predictive of (formal) mathematics achievement. With these findings, the unique role of various forms of memory in the development of mathematics has been addressed and can be taken into account for future research or intervention studies.

### Limitations

Some limitations to this study need to be taken into account when interpreting the findings. Firstly, in this study two subtest of the BAS-3 (i.e., Pattern construction and Word definition) were chosen to control for general intelligence. Nevertheless, it should be mentioned that the Pattern construction subtest has a spatial component. Adding this measure as a covariable might impact the results and underestimate the effect of visuospatial memory in mathematics. Nevertheless, since verbal intelligence was equally taken into account by including Word definition as a covariable, both spatial and verbal skills were controlled for. This could explain why similar significant results were not always found when controlling for both BAS-3 measures and findings including covariables should be interpreted with this in mind.

Second, the use of an episodic verbal long-term memory task was not well suited to test the role of verbal long-term memory in mathematics since arithmetic facts have been shown to be stored in semantic long-term memory (De Smedt, 2016; Whetstone, 1998). The lack of a significant role for verbal long-term memory in mathematics in this study, should thus be interpreted with caution. Third, this study included a standardized mathematics task (TEMA-3), which is comprised of multiple different numerical skills (including counting, arithmetic, word problems, comparison facility, numeral literacy, number facts, and understanding of concepts etc.), but does not include other mathematical skills that are taught in school such as geometry, probability, or measurement. The findings from this study do not generalise to mathematics skills not measured in this study.

## Data accessibility statements

The anonymised data that support the findings of this study and the analysis script including power analysis have been made available on OSF and can be accessed at https://osf.io/jked2.
